# Mediterranean Diet Implementation to Protect against Advanced Lung Cancer Index (ALI) Rise: Study Design and Preliminary Results of a Randomised Controlled Trial

**DOI:** 10.3390/ijerph18073700

**Published:** 2021-04-01

**Authors:** Aristea Gioxari, Dimitrios Tzanos, Christina Kostara, Panos Papandreou, Giannis Mountzios, Maria Skouroliakou

**Affiliations:** 1 Department of Dietetics and Nutritional Science, School of Health Science and Education, Harokopio University, 17671 Athens, Greece; dtzanos86@gmail.com; 2 Department of Nutrition, IASO Hospital, 15123 Athens, Greece; diatrofi@iaso.gr (C.K.); ppapandreou@cibusmed.com (P.P.); 3 Department of Medical Oncology, Henry Dunant Hospital Center, 11526 Athens, Greece; gmountzios@gmail.com

**Keywords:** lung cancer, Mediterranean diet, ALI, platelet count, inflammation, antioxidant vitamins, nutritional status

## Abstract

The Mediterranean diet (MD) has been inversely associated with lung cancer (LC) risk. Hereby we show the preliminary results of our prospective randomised controlled trial in inflammatory and nutritional status of LC patients after 3-month implementation of MD. In total, 30 patients with small-cell or non-small-cell LC (stages III–IV) were enrolled. They were randomly assigned either to Control group, receiving general nutritional guidelines, or the MD group, in which a personalised MD plan was provided. Medical and dietary history, anthropometrics, blood biomarkers, and circulating antioxidant vitamins were assessed. The main outcome was a significantly higher advanced lung cancer inflammation index (ALI) in patients of the control arm than those following MD (*p* = 0.003). In the MD group, platelets were significantly reduced at the study endpoint (*p* = 0.044). BMI and body fat mass remained unchanged in both arms, but serum glucose was significantly higher in control compared to MD group (*p* = 0.017). In conclusion, we showed for the first time that implementing a personalised MD for 3 months is promising to regulate prognostic biomarkers in advanced LC. The final results of our on-going trial will shed a light on the inflammatory, antioxidant and nutritional status of LC patients following MD.

## 1. Introduction

Lung cancer (LC) is now the second most common type of cancer and the main leading cause of death due to malignancies in both men and women worldwide [1]. According to the World Health Organization (WHO), the newly diagnosed LC cases in 2020 reached 2.21 million globally, while 1.80 million deaths caused by LC were reported [1]. It is estimated that by 2035, the number of LC deaths will have increased to 3 million [2]. Lung cancer survival is mostly determined by disease stage and treatment modality, with patients being commonly diagnosed at an advanced stage, either stage III or IV [3,4]. The current 5-year survival rate does not exceed 15%, and ranges from approximately 70% for early stage cancer (stage I or II) to 1–5% for distant metastases [2,3,4].

The etiology of LC is rather complex. Tobacco smoking is an important risk factor for LC development, while incidence and mortality of LC are tightly linked to socio-demographic characteristics [5]. The burst in economic development, together with the initiation of tobacco control policies, resulted in decline of LC incidence and mortality in the industrialised world since the 1990s, with a more profound effect in men than women [5,6]. This discrepancy has been attributed to the different background in tobacco use between the two sexes [7]. Similar trends have been observed in the developing world, but later as compared with the industrialised countries [5]. Limited access to healthcare leading to delayed diagnosis and treatment, lack of tobacco control programs, environmental contamination, and sociocultural barriers contribute to the increase of LC incidence and mortality [8].

LC is quite heterogeneous and is classified into the Small Cell LC (SCLC) and the Non-Small Cell LC (NSCLC). NSCLC represents 85% of all LC and includes the squamous cell, the adenocarcinoma, and other histotypes [9,10]. Chronic inflammation, oxidative stress, protease–antiprotease imbalance, and disturbed repair mechanisms are well documented in LC [10]. LC patients report decline in physical functioning, increased pain, and generally low quality of life [11]. Main symptoms are fatigue, loss of appetite, and weight loss, as well as dyspnea, hemoptysis, and chest pain [12]. Treatment, which depends strictly on the Tumor-Nodes-Metastasis (TNM) Classification of the primitive neoplasm, involves surgery for resectable disease combined with radiotherapy and chemotherapy for locally advanced/metastatic disease [12]. During the last years, new insights into the LC molecular mechanisms have brought about novel treatment strategies, which are generally more effective and better tolerated than standard treatment [12]. To this point, an adequate nutritional status may enhance physical functioning and treatment efficiency and improve somatic symptoms and psychological distress, thus overcoming the burden of malnutrition [13,14].

In fact, inadequate energy intake, metabolic alterations, depression, fatigue, and chemotherapy-induced toxicity, have been reported to lead to skeletal muscle mass loss and systemic inflammation syndrome, in which acute phase proteins, such as C-reactive protein (CRP), serum albumin levels (ALB), and white cell count are altered [15]. Lower ALB has been associated with a higher incidence of systemic inflammation and a worse overall survival [9,15]. Additionally, a higher CRP concentration and a greater Neutrophil to Lymphocyte Ratio (NLR) have been related to a worse overall survival in LC [9,15,16]. An adequate protein and energy intake represents the first target of a correct nutritional intervention in cancer patients [15].

What is more, diet has been shown to improve quality of life in cancer survivors [9]. Our research team has recently shown that a greater adherence to the Mediterranean Diet (MD) was associated with greater circulating antioxidant vitamins (i.e., vitamin C, retinol and a-tocopherol), lower body mass index (BMI) and body fat mass (BFM), as well as improved blood glucose levels in women recently diagnosed with breast cancer [17]. The traditional MD is characterised by high consumption of vegetables, legumes, fruits, fish, nuts, whole grains including non-refined cereals and olive oil, low-to-moderate consumption of minimally processed dairy products, regular but moderate intake of wine during meals, and low consumption of red meat, poultry, and highly processed and energy-dense foods rich in saturated fatty acids and sugar [18]. In 2010, the MD was included on the list of the intangible cultural heritage of humanity of United Nations Educational, Scientific, and Cultural Organization (UNESCO) [19].

It is documented that the MD has been inversely associated with LC risk [20,21], but to date, data regarding the effects of MD on the nutritional status of patients with LC are scarce. This study shows the design of this prospective randomised controlled trial and the preliminary results on the inflammatory and nutritional status of LC patients with advanced LC (stages III or IV), after 3-month implementation of a personalised MD.

## 2. Materials and Methods

### 2.1. Ethics

The Ethics Committee of “IASO Hospital” (Athens, Greece) reviewed and approved the study protocol (Approval Code #31052019C). The trial was conducted according to the principles of Helsinki Declaration (1964) and its later amendments, and was in line with terms of Good Clinical Practice. All laboratory techniques were standardized and the staff was properly trained.

### 2.2. Participants

Adult LC outpatients, who were monitored at IASO Hospital, were enrolled in the study. Recruitment took place between January 2018 and April 2019. Patients who agreed to participate were provided with a detailed information leaflet describing the aims, methods, benefits, and potential hazards of the study. Each recruited patient provided a written informed consent, and a copy of the signed consent was kept by the patient.

Inclusion criteria: Adult men and women (≥18 years of age) with histological or cytological LC diagnosis at stages III–IV of both histological types (SCLC or NSCLC) were included in the trial. Additional criteria were conventional treatment during the trial (i.e., chemotherapy, radiotherapy, immunotherapy, molecular therapy, surgery >3 months, or a combination of the above) and the ability to ambulate independently, which corresponded to scoring 0 or 1 of the Eastern Cooperative Oncology Performance Status (ECOG PS) [22].

Exclusion criteria: History of any other malignancy ≤5 years; undergone surgery ≤3 months prior to screening; medical conditions (e.g., obstructive ileus) that would interfere with the study protocol; malabsorption; serious chronic diseases (e.g., advanced heart, liver or renal failure, congenital metabolic diseases); active infection; diagnosis of severe psychiatric illness; alcoholism or drug use; pregnant or lactating women; vitamin or inorganic supplement use ≤6 months prior to screening; following vegan or macrobiotic diet ≤5 years prior to screening; using weight loss medications; or having unregulated hyperthyroidism, hypothyroidism or other endocrine disorder that affects metabolism.

### 2.3. Study Design

In this two-armed, single center, randomised, controlled, 3-month pilot trial, LC patients were randomly assigned either to the Control or the Intervention arm. Simple randomisation was chosen and the randomisation sequence was computer generated by an independent statistician. After randomisation, the statistician sent the randomisation list to the trial principal investigator who completed a participant form for each subject, including the treatment and the patient trial number and put it in a sealed envelope. Blinding of the allocated treatment was maintained to the data analyst and was exposed only after the assessment of outcomes.

The Intervention group (MD group) received tailor-made personalised dietary intervention by experienced dietitians. More specifically, individual counselling was scheduled every 15 days providing daily dietary plans (specific meals, products, recipes and food portions in grams), coupled with educational booklets, food diaries, and individual nutritional advice. The MD group also received the updated American Cancer Society Guidelines on Nutrition and Physical Activity for Cancer Prevention [23]. Daily energy needs were determined according to the basic metabolic rate equation of Harris–Benedict and physical activity levels, compensating for weight or skeletal muscle mass loss. Nutritional guidance was based on the Mediterranean dietary pattern and patients were counseled to consume: (1) olive oil instead of other plant-based oils on a daily basis, (2) whole grains and non-refined cereals daily, (3) two to three cups per day of herbal teas due to their high content in phytochemicals [24], (4) seasonal fruits and vegetables with high antioxidant capacity (i.e., broccoli, grapes, pomegranate, strawberries, cauliflower, blueberries, and blackberries) daily [25], and (5) fish, legumes, and eggs at least once per week. Patients in the MD group were also trained on meal preparation techniques such as cooking without added salt, sugar, or butter, and promoted the use of traditional Mediterranean herbs, such oregano, rosemary, etc. Counselling was centered on the daily distribution of nutrients in relation to the total caloric value as follows: 1–1.5 g per kg of body weight as protein, about 30% of the total energy as fat (<10% as SFAs, ∼10% as MUFAs, and ∼10% as PUFAs), the remaining energy % as carbohydrate, and 20–30 g fiber per day. Some health problems related to the participants’ medical history (e.g., constipation, esophageal reflux) were taken into consideration to the dietary intervention.

In the Control arm, regular 15-day phone interviews were scheduled to provide general nutritional guidelines based on the updated American Cancer Society Guidelines on Nutrition and Physical Activity for Cancer Prevention [23]. In total, three personal appointments were conducted with each patient; at the beginning of the study, at 2 months, and at the end of the study (3 months).

### 2.4. Screening

Medical history: The appointed oncologist recorded medical history, including general information (age, sex, smoking history), as well as disease specific data (i.e., disease stage, cancer histological type, age of diagnosis, symptoms and complications, as well as treatment regimen).

Dietary assessment: Experienced dietitians conducted face-to-face interviews with the enrolled patients. Dietary intake was evaluated using a semi-quantitative Food Frequency Questionnaire (FFQ) validated in Greek cancer patients [26], at baseline and follow up (3 months). The FFQ was used to assess the intake of foods and beverages commonly consumed in Greece. Frequency of consumption was determined as the number of times a food was consumed within a month, in small, medium, or large portion sizes. During the interviews, portion food models and pictures were demonstrated to facilitate the estimation of portion sizes. We confirmed adherence to MD based on food diaries, which were provided during the scheduled counselling sessions by each patient every 15 days. Patients of the Control arm sent the food diaries by e-mail. Patients who provided food records for less than three days per week (2 weekdays and 1 weekend day) were excluded from final analysis. The degree of MD adherence was estimated by the MD score, which relied on the dietary components frequently consumed in the traditional MD, namely non-refined cereals, fruits, vegetables, legumes, olive oil, fish, and potatoes [27]. Higher values of the MD score indicated a greater MD adherence. Diet Analysis Plus (version 6.1, Wadsworth 2003) was used to analyse and evaluate dietary plans, FFQs, and food diaries. In order to assess consumption of commercially available foods and fortified food products not included in the software, nutritional labels on food packaging were recorded and registered in the software package.

Performance status and physical activity: The Eastern Cooperative Oncology Group (ECOG) performance status (PS) was applied to assess ambulatory capacity [22]. ECOG PS has five levels ranging from 0 (fully ambulatory without symptoms) to 4 (completely disabled). Patients with an ECOG PS of 0 and 1 are labelled as having “good” PS for clinical research purposes [22]. The “International Physical Activity Questionnaire” was used to assess physical activity levels [28]. It takes into account both the daily activities e.g., household tasks, as well as organised physical exercise performed during leisure time. Classification of physical activity was based on the concept of metabolic equivalent (MET). One MET is equal with resting metabolic rate, while the intensity of physical activity was expressed as a multiple of MET.

Blood sample collection: After overnight fasting, standard blood withdrawal (20 mL) was performed for each participant (10 mL for serum and 10 mL for plasma isolation) both at baseline and follow up (3 months). For plasma isolation, EDTA was used as anticoagulant. To collect serum, whole blood was previously allowed to clot at room temperature for 20 min. Whole blood samples were centrifuged at 3000 rpm for 10 min at 4 °C. For all blood analyses, freshly drawn samples were used.

Anthropometrics: Body weight (BW), body fat mass (BFM), and percentage BFM were measured for both groups at the beginning and at the end of the study (3 months) with the method of Air Displacement Plethysmography for research and clinical applications (BOD POD^®^ Body Composition Tracking Systems, Life Measurement, Inc., Rome, Italy). Two hours before measurements, participants had not performed exercise, had not consumed any foods or drinks, and they were dressed only in their underwear. Height was also measured at the beginning of the study with a calibrated stadiometer to the nearest 0.1 cm. Body mass index (BMI) was calculated for every patient as the ratio of weight (kg) to the square of height (m^2^).

Blood analyses: Blood analyses followed the criteria of the World Health Organization Reference Laboratories. Haematology analysis was performed using an automatic analyser (DxH 800 analyser, Beckman Coulter Inc., Nyon, Switzerland). Serum glucose, total cholesterol, high-density lipoprotein (HDL), low-density lipoprotein (LDL), triacylglycerols, albumin, and C-reactive protein (CRP) were quantified with an automatic biochemical analyser using manufacturer’s reagents (Cobas 8000 modular analyser, Roche Diagnostics GmbH, Mannheim, Germany). Advanced lung cancer inflammation index (ALI) was also calculated (BMI × Alb/neutrophil lymphocyte ratio) for each participant as a marker of disease prognosis [29].

A reversed-phase high performance liquid chromatography (HPLC) system (model 1050, Agilent Technologies, Waldbronn, Germany) coupled with ultraviolet (UV) and fluorescence (FL) detectors, quaternary pump, auto-sampler, and data analysis software, was used to quantify antioxidant vitamins A (retinol), E (alpha-tocopherol) and C (ascorbic acid) in plasma samples, according to previously published methods [30,31]. With regard to vitamin C, fresh plasma samples were extracted with an equal volume of 10% metaphosphoric acid and the acidic extracts were separated on a Synergy Polar-RP column (150 mm × 4.6 mm, 4 μm particle size, Merck-Millipore, Darmstadt, Germany), using 50 mM potassium phosphate buffer at pH 2.5 as mobile phase. For quantification of vitamins A and E, the internal standard (6 μmol/L retinyl acetate) was added in plasma samples followed by extraction with an equal volume of ethyl acetate-butanol mixture of (1:1). Organic extracts were separated on a fully end-capped C18 (150 mm × 4.6 mm, 5 μm particle size, Merck-Millipore, Darmstadt, Germany) using 100% methanol as mobile phase. Chromatographic runs were performed isocratically at a flow rate of 1.0 mL/min and the column temperature was kept constant at 28 °C. The eluted compounds were detected spectrophotometrically at 245 nm (vitamin C), 292 nm (vitamin E) and 325 nm (retinyl acetate) and fluorometrically at 325 nm/475 nm excitation/emission wavelengths (retinyl acetate). Sample concentrations were calculated from peak areas by a linear calibration model. All vitamin standards used were of HPLC-grade (Sigma-Aldrich, Steinheim, Germany) and the water used was ultrapure (Milli-Q water filtration system, Millipore Spa, Rome, Italy). All solvents were of HPLC-grade and were purchased from Merck (Darmstadt, Germany). Vitamin C deficiency was defined as circulating ascorbic acid <1.94 mg/L (or <11 μmol/L) [32], vitamin A as circulating retinol <9.80 μg/dL (or <0.35 µmol/L) [33] and vitamin E as circulating alpha-tocopherol <5.17 mg/L (or <12 µmol/L) [34].

### 2.5. Statistical Analysis

All analyses were performed with the SPSS statistical software (version 21.0, SPSS, Inc, ΙΒΜ, Chicago, IL, USA). Descriptive statistics were calculated for all parameters. Continuous data were expressed as medians and ranges (non-Gaussian distribution, Wilk test) and dichotomous variables as counts. Comparisons between the two study groups were performed using the Student’s *t* test or the Mann–Whitney test. For investigating possible intra-group differences, a paired samples *t*-test or the Wilcoxon test was applied. Statistical significance was set at *p*-value < 0.05.

Our primary outcome was a significant reduction of the ALI index in LC patients following a personalised MD compared to control group. Based on ALI outcome, we conducted power size calculation with estimated power of 80% and a level of significance of 5% (two sided) to determine the population size of our on-going prospective randomised controlled trial.

## 3. Results

### 3.1. Participants

**Figure 1:**
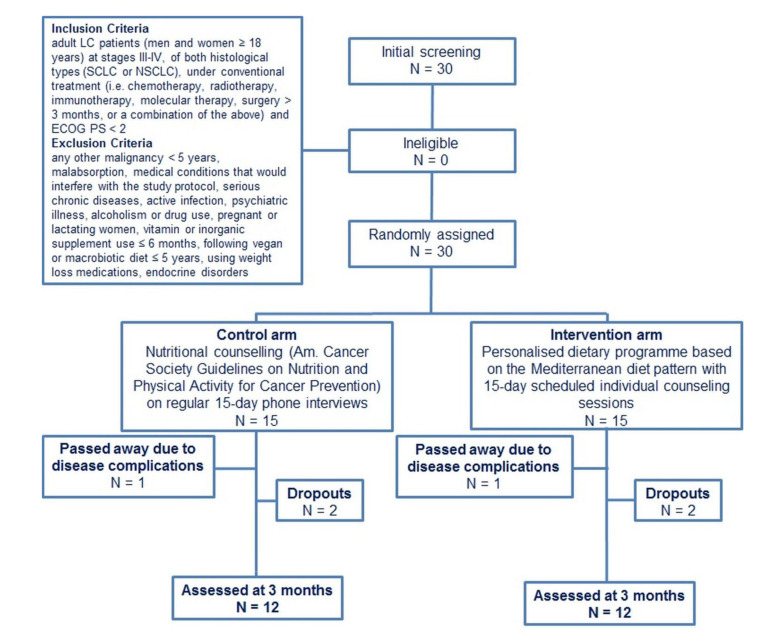
Study’s flowchart. LC, lung cancer; SCLC, small cell lung cancer; NSCLC, non-small cell lung cancer; ECOG PS, Eastern Cooperative Oncology Group performance status.

A total of 30 patients with advanced LC met the criteria and were included in the study (Figure 1). Their nationality was Greek and they were residents of Attica in Greece. General characteristics of enrolled LC patients at baseline are shown in Table 1. The majority of participants suffered from NSCLC (27 out of 30) at stage IV (25 out of 30), while 23 patients had unoperated metastatic cancer. Two patients were underweight, while 7 were obese. Most patients (25 out of 30) were fully ambulatory (ECOG PS equal to 0) and 13 patients were current smokers. All volunteers had been on a diet (at least once) in the past.

In total, 6 patients did not complete the follow up assessment at three months due to impairment of their clinical condition (*n* = 4) or death (*n* = 2). As a result, 12 patients in control and 12 patients in MD group completed the trial. At initiation of the study protocol, there were no significant differences in anthropometrics, blood indices, and dietary intake between the two LC arms (Table 1).

### 3.2. Anthropometrics and Physical Activity

The BMI of enrolled patients did not change at follow up (3 months) compared to baseline neither in control (*p* = 0.133) nor in the MD group (*p* = 0.916) (Table 2). Similarly, body fat mass percentage remained the same throughout the trial in both study groups (*p* = 0.514 and *p* = 0.665, respectively). Physical activity levels of the MD arm had a tendency to increase, but change was insignificant (*p* = 0.053) at the study endpoint. In the control arm, physical activity remained unchanged (*p* = 0.128) during the trial.

### 3.3. Blood Markers

With regard to prognostic and inflammatory biomarkers markers (Table 2), neutrophil to lymphocyte ratio (NLR) and CRP levels remained unchanged from the beginning to the end of the study for both groups (*p* = 0.174 and *p* = 0.193 respectively). On the other hand, ALI of the control arm was significantly increased at follow up compared to baseline (78.4 ± 30.6 vs. 55.0 ± 27.1 respectively, *p* = 0.025) and this increase remained significant when compared to the MD group (*p* = 0.003). In addition, platelet count of the MD group was significantly reduced at the study endpoint compared to baseline (*p* = 0.044).

Serum glucose levels in the control arm tended to increase during the intervention period, without reaching a statistical significance (*p* = 0.059). Nevertheless, serum glucose was significantly higher in control compared to the MD group (*p* = 0.017) at the trial endpoint. Albumin levels remained unchanged from the beginning to the end of the study for both groups (*p* = 0.095).

### 3.4. Dietary Intake and Circulating Vitamins

In the MD group, MD score, dietary intakes of monounsaturated fatty acids (MUFAs) and crude fibers were significantly higher, and the intake of saturated fatty acids (SFAs) was significantly lower at the end compared to initiation of the study (*p* = 0.004, *p* = 0.002, *p* = 0.005 and *p* = 0.006 respectively) (Table 2). With regard to antioxidant vitamins, consumption of vitamin C in the MD group was significantly elevated (*p* = 0.024) at 3 months compared to baseline, and this increase remained significant when compared to control group (*p* = 0.004). Nevertheless, this increase did not affect plasma vitamin C concentration (*p* = 0.402). Moreover, intake of alpha-tocopherol (vitamin E) and retinol active equivalents (vitamin A) were the same throughout the trial in both groups. Current smokers decreased by 40% in the control and by 50% in the intervention arm.

### 3.5. Sample Size Calculation

Based on ALI results (primary outcome) our on-going randomised controlled trial would require a sample size of 48 (i.e., 24 for each group, assuming equal group sizes) to achieve a power of 80% and a level of significance of 5% (two sided). Taking into account a dropout about 30–35%, the required sample size rises to 64.

## 4. Discussion

To the best of our knowledge, this is the first randomised controlled trial evaluating the effects of implementing Mediterranean diet (MD) on nutritional status and disease related markers in patients with lung cancer (LC). Taking into account energy and protein needs for cancer patients, a personalised MD plan was provided and individual counselling sessions were scheduled on a regular basis. The main outcome of this 3-month intervention was a significantly higher advanced lung cancer inflammation index (ALI) in patients of the control group than those following MD. During the trial, platelet count, another prognostic biomarker of LC, was significantly reduced in the MD group.

It is well documented that inflammation in the tumor microenvironment determines tumor proliferation, angiogenesis, invasion, metastasis and survival [35]. To date, several systemic inflammatory factors, including C-reactive protein (CRP), hypoalbuminaemia, neutrophil-to-lymphocyte ratio (NLR), platelet count, and advanced lung cancer inflammation index (ALI), have been demonstrated to have a prognostic value in LC [29,36,37]. In fact, low ALI has been correlated with poor prognosis in patients with NSCLC or SCLC who underwent surgery, while optimal cutoff values were estimated at 37.66 and 48.2 respectively [37,38]. There is evidence that ALI is superior to other inflammation-based prognostic scores [37]. However, the specific mechanism for the predictive value of this scoring system is uncertain [39]. Systemic inflammation, besides promoting tumor growth, is responsible for many cancer-related symptoms including cancer cachexia and anorexia [40]. To this point, ALI estimates systemic inflammation taking into account two different systemic inflammatory indices, ALB and NLR, together with BMI as a marker of nutritional status [39]. According to the preliminary results of our study, ALI of the control arm was significantly higher at follow up (3 months) compared to baseline (*p* = 0.025), while it remained stable in LC patients following MD. This ALI increase in the control group remained significant when compared to the MD group at the trial endpoint (*p* = 0.003). Consequently, MD protects against ALI rise in LC. Platelet count is another prognostic biomarker in LC, playing a significant role in cancer cell growth, progression, and metastasis [36]. Hypercoagulability is a sign of a more aggressive disease, while a thromboembolism is one of the major causes of mortality in cancer patients [36]. In our study, platelets were significantly reduced in patients following MD at the end compared to beginning of the study (*p* = 0.044), strengthening our observation that MD exerts beneficial effects on lung cancer patients.

Vitamins with antioxidant activity have received much attention in LC. Vitamins A, C, and E have been shown to possess a protective role against lung cancer risk, but with a modification in effects by the intensity of cigarette exposure [41]. During our trial, dietary intake of vitamin C increased significantly by approximately 72 mg/day (*p* = 0.024) in the MD group. Accordingly, circulating vitamin C tended to increase in the MD arm but did not reach statistical significance. This could be explained by the increased oxidative injury and quick depletion of endogenous antioxidant mechanisms observed in lung cancer patients [42]. In addition, cigarette smoking can affect blood levels of vitamin C. Alberg [43] reported that circulating concentrations of vitamin C decreased with increasing numbers of cigarettes smoked per day. To this point, 17% of LC patients in the MD group of our study were still smokers.

Cachexia (weight loss with altered body composition) is a multifactorial syndrome propagated by symptoms that impair caloric intake, tumor byproducts, chronic inflammation, altered metabolism, and hormonal abnormalities [44]. Loss of weight in cancer patients cannot be easily reversed by standard nutritional interventions, while treatment intended to normalise underlying metabolic abnormalities is critical for efficient nutrient utilization. [44]. Therefore, another important outcome in the present study was the maintenance of BMI and body composition in both study groups. Insulin resistance has been widely recorded in malignant tumors contributing to cancer cachexia due to chronic exposure to pro-inflammatory cytokines, such as TNF-a, IL-6, and insulin growth factor binding protein [45]. In our study, serum glucose levels were also maintained in the MD arm throughout the trial, but this was not the case for the control group. Serum glucose was significantly enhanced in control compared to the MD group at the study endpoint (*p* = 0.017).

Due to the small sample size, the high dropout, and the multiple comparisons between the two groups, the overall power of analysis was low. We are also aware of the limitations existing in the use of control arms in nutritional intervention studies, e.g., poor adherence of participants in the control arm or difficulty of applying homogenous advice across the participants within the control group [46]. Nevertheless, based on the results of our primary outcome (ALI) and considering a dropout about 30–35%, the sample size needed to strengthen the results of our on-going prospective randomised control trial is 64 (80% power, 5% level of significance and assuming equal group sizes).

## 5. Conclusions

In conclusion, we showed for the first time that the implementation of an individualised Mediterranean dietary plan for 3 months has promising results in regulating the advanced lung cancer inflammation index (ALI), platelet count, and glycemic profile of patients with advanced lung cancer (stages III–IV). The final results of our on-going prospective randomised controlled trial will shed a light on the effects of Mediterranean diet on the inflammatory, antioxidant, and nutritional status of advanced lung cancer patients.

## Figures and Tables

**Table 1:** Characteristics of enrolled LC patients at initial screening.

Characteristics	Enrolled Patients (*N* = 30)	Control Group (*N* = 15)	MD Group (*N* = 15)	*p*-Value
Sex Males Females	16 14	8 7	8 7	-
Age (years)	52.2 ± 27.3	52.9 ± 27.8	51.5 ± 27.7	0.891
BMI (kg/m^2^) <18.5 18.5–24.9 25–29.9 >30	26.8 ± 5.5 2 9 12 7	27.4 ± 4.1 0 5 7 3	26.2 ± 6.6 2 4 5 4	0.550 -
BFM (Kg)	27.8 ± 10.7	28.6 ± 9.1	26.9 ± 12.3	0.688
% BFM	37.0 ± 9.2	37.4 ± 7.3	36.7 ± 11.0	0.864
LC type SCLC NSCLC	3 27	2 13	1 15	-
LC stage III IV	5 25	2 13	3 12	-
Smoking Yes In the past Never	13 16 1	6 8 1	7 8 0	-
Cigarettes per day by current smokers 1–5 6–14 15–24 25–34	13 4 4 3 2	2 2 1 1	2 2 2 1	-
LC treatment Surgery (>3 months) Unoperated Combined chemo- radiotherapy Chemotherapy Immunotherapy Combined chemo- immunotherapy Molecular therapy	7 23 12 3 4 3 1	2 13 5 1 3 3 1	5 10 7 2 1 0 0	-
ECOG performance status Score 0 Score 1	25 5	13 2	12 3	-
METS-min/week	288.6 ± 106.7	287.1 ± 90.8	290.1 ± 123.8	0.940
Hematocrit (%)	38.2 ± 5.0	38.9 ± 5.3	37.4 ± 4.8	0.447
Hemoglobin (g/dL)	12.4 ± 1.8	12.7 ± 1.8	12.2 ± 1.7	0.438
White blood cell (K/μL)	5.3 × 10^3^ ± 3.4 × 10^3^	5.5 × 10^3^ ± 3.3 × 10^3^	5.1 × 10^3^ ± 3.6 × 10^3^	0.766
Neutrophils (%)	62.4 ± 12.6	64.3 ± 13.2	60.3 ± 11.9	0.398
Lymphocytes (%)	25.6 ± 10.8	23.6 ± 11.4	27.8 ± 10.0	0.301
NLR	3.2 ± 2.1	3.8 ± 2.6	2.5 ± 1.1	0.100
Platelets (K/μL)	241.5 × 10^3^ ± 94.9 × 10^3^	256.1 × 10^3^ ± 76.1 × 10^3^	226.8 × 10^3^ ± 111.4 × 10^3^	0.407
Glucose (mg/dL)	103.8 ± 20.6	99.9 ± 16.4	107.9 ± 24.2	0.304
Total cholesterol (mg/dL)	201.5 ± 40.1	209.1 ± 42.0	193.4 ± 37.6	0.300
HDL (mg/dL)	53.3 ± 17.5	55.2 ± 20.5	51.3 ± 14.1	0.562
LDL (mg/dL)	126.8 ± 33.3	134.9 ± 31.1	118.1 ± 34.4	0.179
Triacylglycerols (mg/dL)	132.8 ± 96.3	141.3 ± 116.6	122.8 ± 69.3	0.622
Albumin (g/dL)	4.2 ± 0.4	4.1 ± 0.3	4.2 ± 0.4	0.630
Vitamin A (mg/L)	0.5 ± 0.3	0.5 ± 0.2	0.6 ± 0.3	0.579
Vitamin C (mg/L)	6.0 ± 2.4	5.5 ± 2.7	6.5 ± 2.0	0.258
Vitamin E (mg/L)	13.1 ± 4.8	13.2 ± 4.5	13.1 ± 5.3	0.977
CRP (mg/L)	11.2 ± 13.6	12.3 ± 13.4	10.1 ± 14.3	0.667
ALI	47.2 ± 29.9	45.0 ± 31.8	49.3 ± 28.9	0.704
MD score	30.8 ± 4.8	31.7 ± 4.8	29.9 ± 4.7	0.311
Sugars (g/day)	60.8 ± 23.1	56.5 ± 24.5	65.2 ± 21.5	0.314
SFAs (g/day)	30.5 ± 9.5	27.6 ± 7.2	33.3 ±10.8	0.101
PUFAs (g/day)	13.6 ± 8.0	13.7 ± 5.9	13.6 ± 9.8	0.975
MUFAs (g/day)	24.3 ± 10.2	27.2 ± 12.1	21.5 ± 7.2	0.127
Fibers (g/day)	4.0 ± 2.7	4.4 ± 3.1	3.5 ± 2.2	0.385
Vitamin C (mg/day)	80.5 ± 42.6	88.4 ± 37.7	72.6 ± 46.8	0.319
Vitamin A RAE (mcg/day)	596.8 ± 698.6	590.1 ± 566.9	603.5 ± 830.3	0.959
alpha-tocopherol (mcg/day)	7.4 ± 2.7	7.4 ± 2.5	7.3 ± 3.0	0.968

Data are expressed as counts or mean values ± standard deviation of mean (SD). *p*-value: significant differences between the control and the intervention group at baseline analysed by independent sample *t*-test or the Mann–Whitney test, where applicable. Difference was considered significant at *p* < 0.05. LC, lung cancer; MD, Mediterranean diet; BMI, body mass index; BFM, body fat mass; NLR, neutrophil to lymphocyte ratio; HDL, high density lipoprotein; LDL, low density lipoprotein; CRP, C-reactive protein; ALI, advanced lung cancer inflammation index; RAE, retinol active equivalents

**Table 2:** Anthropometrics, biochemical parameters and vitamin intake of enrolled lung cancer (LC) patients who completed the trial at baseline and follow up (3 months).

Parameter	Group	Baseline (*N* = 12)	3 Months (*N* = 12)	*p*-Value	* *p*-Value
		Mean ± SD	Mean ± SD		
BMI (kg/m^2^) <18.5 18.5–24.99 25–30 >30	control	27.5 ± 4.7 0 5 4 3	25.7 ± 3.1 0 5 5 2	0.133 - - - -	0.138
	MD	26.3 ± 6.9 1 (8.3) 4 (33.3) 4 (33.3) 3 (25)	26.4 ± 6.4 1 (8.3) 5 (41.7) 4 (33.3) 2 (16.7)	0.916 - - - -	
Body fat mass (kg)	control	26.2 ± 7.3	26.7 ± 8.1	0.446	0.401
	MD	26.0 ± 12.8	26.2 ± 11.2	0.818	
% body fat mass	control	36.4 ± 7.4	36.8 ± 7.2	0.514	0.366
	MD	34.7 ± 10.5	35.4 ± 8.0	0.665	
METS-min/week	control	283.8 ± 97.5	323.0 ± 84.2	0.128	0.711
	MD	298.2 ± 112.4	350.0 ± 109.6	0.053	
Current smokers	control	5 (41.7)	3 25.0)	-	-
	MD	6 (50)	2 (16.7)	-	-
Hematocrit (%)	control	39.4 ± 4.2	39.2 ± 4.4	0.795	0.477
	MD	37.0 ± 5.0	38.0 ± 3.1	0.509	
Hemoglobin (g/dL)	control	12.8 ± 1.4	12.7 ± 1.5	0.545	0.311
	MD	12.0 ± 1.7	12.3 ± 1.2	0.422	
White blood cell (K/μL)	control	4.6 × 10^3^ ± 3.0 × 10^3^	3.4 × 10^3^ ± 2.8 × 10^3^	0.124	0.144
	MD	5.2 × 10^3^ ± 3.9 × 10^3^	6.0 × 10^3^ ± 3.2 × 10^3^	0.482	
Neutrophils (%)	control	61.1 ± 12.7	58.8 ± 13.0	0.460	0.158
	MD	62.2 ± 9.4	65.0 ± 9.9	0.150	
Lymphocytes (%)	control	26.8 ± 10.6	29.9 ± 13.0	0.299	0.088
	MD	26.8 ± 8.8	24.2 ± 9.8	0.090	
NLR	control	2.9 ± 2.1	2.8 ± 2.2	0.723	0.174
	MD	2.6 ± 1.0	3.2 ± 1.6	0.071	
Platelets (K/μL)	control	252.7 × 10^3^ ± 74.8 × 10^3^	214.0 × 10^3^ ± 164.8 × 10^3^	0.372	0.441
	MD	256.9 × 10^3^ ± 96.1 × 10^3^	175.3 × 10^3^ ± 133.1 × 10^3^	**0.044**	
Glucose (mg/dL)	control	100.3 ± 17.1	114.6 ± 34.5	0.059	**0.017**
	MD	108.7 ± 26.2	104.4 ± 18.1	0.368	
Cholesterol (mg/dL)	control	207.8 ± 42.8	209.3 ± 38.5	0.898	0.394
	MD	185.7 ± 34.5	200.7 ± 32.7	0.183	
HDL (mg/dL)	control	58.1 ± 21.5	51.6 ± 17.7	0.066	0.093
	MD	50.8 ± 15.1	52.8 ± 14.3	0.600	
LDL (mg/dL)	control	132.8 ± 26.0	130.1 ± 26.7	0.753	0.302
	MD	110.4 ± 29.4	119.3 ± 25.7	0.229	
Triacylglycerols (mg/dL)	control	108.7 ± 43.4	148.6 ± 113.1	0.113	0.409
	MD	125.9 ± 71.4	143.3 ± 85.7	0.225	
Albumin (g/dl)	control	4.2 ± 0.3	4.1 ± 0.4	0.435	0.095
	MD	4.2 ± 0.4	4.4 ± 0.6	0.142	
Vitamin A (mg/L)	control	0.5 ± 0.1	0.5 ± 0.2	0.886	0.274
	MD	0.6 ± 0.3	0.5 ± 0.1	0.223	
Vitamin C (mg/L)	control	5.9 ± 2.8	5.2 ± 4.4	0.693	0.442
	MD	6.4 ± 2.0	7.2 ± 3.8	0.402	
Vitamin E (mg/L)	control	13.5 ± 4.8	14.9 ± 3.2	0.402	0.630
	MD	13.3 ± 5.4	13.7 ± 5.7	0.768	
CRP (mg/L)	control	9.4 ± 11.7	11.9 ± 18.3	0.687	0.193
	MD	11.6 ± 14.9	3.8 ± 4.1	0.116	
ALI	control	55.0 ± 27.1	78.4 ± 30.6	**0.025**	**0.003**
	MD	48.2 ± 20.9	45.2 ± 18.7	0.542	
MD score	control	31.6 ± 3.2	33.1 ± 5.1	0.163	**0.031**
	MD	29.2 ± 5.9	34.7 ± 3.7	**0.004**	
Sugars (g/d)	control	56.8 ± 25.8	55.0 ± 22.9	0.835	0.980
	MD	69.0 ± 21.2	67.5 ± 18.2	0.848	
SFAs (g/d)	control	27.6 ± 7.1	23.1 ± 8.7	0.172	0.279
	MD	35.0 ± 11.3	26.2 ± 11.5	**0.006**	
PUFAs (g/d)	control	13.4 ± 6.5	11.7 ± 6.7	0.398	0.838
	MD	15.0 ± 11.1	14.2 ± 8.1	0.832	
MUFAs (g/d)	control	27.1 ± 14.2	27.9 ± 13.9	0.858	0.102
	MD	21.4 ± 8.5	30.4 ± 5.8	**0.002**	
Fibers (g/d)	control	3.9 ± 3.3	3.8 ± 2.5	0.930	**0.025**
	MD	4.1 ± 2.3	8.5 ± 4.0	**0.005**	
Vitamin C (mg/day)	control	85.2 ± 44.1	50.7 ± 33.3	0.097	**0.004**
	MD	67.6 ± 52.4	139.2 ± 63.7	**0.024**	
Vitamin A RAE (mcg/day)	control	789.2 ± 906.3	356.1 ± 663.9	0.655	0.872
	MD	490.5 ± 646.0	581.3 ± 430.3	0.559	
alpha-tocopherol (mcg/day)	control	7.7 ± 3.4	6.2 ± 2.4	0.124	0.497
	MD	7.8 ± 2.6	7.5 ± 5.2	0.898	

Data are expressed as counts or mean values ± standard deviation of mean (SD). *p*-value: comparison with the baseline values by the paired samples *t* test or the Wilcoxon test, where applicable; difference was considered significant at *p* < 0.05. * *p*-value: significant differences in changes at endpoint between the two groups applying the independent samples *t*-test or the Mann–Whitney test, where applicable; difference was considered significant at *p* < 0.05; significant p-values are marked bold in the table. LC, lung cancer; MD, Mediterranean diet; BMI, body mass index; BFM, body fat mass; NLR, neutrophil to lymphocyte ratio; HDL, high density lipoprotein; LDL, low density lipoprotein; CRP, C-reactive protein; ALI, advanced lung cancer inflammation index; RAE, retinol active equivalents.
